# 44-year-old Man with Hemoptysis and Hypoxemic Respiratory Failure: A Case Report

**DOI:** 10.5811/cpcem.1418

**Published:** 2023-05-30

**Authors:** Bryan P. McNeilly, Dominic M. Williams, Laura J. Bontempo, J. David Gatz

**Affiliations:** * University of Maryland Medical Center, Department of Emergency Medicine, Baltimore, Maryland; † CarolinaEast Medical Center, Department of Emergency Medicine, New Bern, North Carolina; ‡ University of Maryland School of Medicine, Department of Emergency Medicine, Baltimore, Maryland

**Keywords:** Clinicopathological cases, infectious disease, hemoptysis

## Abstract

**Introduction:**

Hemoptysis can be a highly alarming presentation in the emergency department (ED). Even seemingly minor cases may represent potentially lethal underlying pathology. It requires thorough evaluation and careful consideration of a broad differential diagnosis.

**Case Presentation:**

A 44-year-old man presented to the ED with a concern of hemoptysis in the setting of recent fever and myalgias.

**Discussion:**

This case takes the reader through how to approach the differential diagnosis and diagnostic work-up of hemoptysis in the ED setting and then reveals the surprising final diagnosis.

## CASE PRESENTATION (DR. MCNEILLY)

A 44-year-old man with a history of polysubstance abuse and homelessness presented to an urban emergency department (ED) in Baltimore, Maryland, with hemoptysis. Symptoms started one week prior to arrival, with three days of fevers and myalgias. On day four, he developed nausea, diarrhea, and a dry cough. His cough became progressively worse over the following days, and on day seven he developed hemoptysis, which prompted his visit to the ED. He denied leg swelling, abdominal pain, dysuria, hematuria, arthralgias, rashes, wounds, dizziness, numbness, and headaches. The patient also denied any sick contacts, including tuberculosis or coronavirus disease 2019 (COVID-19) exposures.

The patient had no known past medical or surgical history and did not use any medications. He stated that he had been homeless for several months and had been living on the streets despite the cool weather. He stated that he drank approximately six beers per day and smoked cigarettes. He occasionally smoked crack cocaine and marijuana. The last time he had used either substance was several weeks prior to his presentation. He also stated that he smoked methylenedioxy-methylamphetamine (MDMA), most recently the morning prior to his arrival.

The patient’s vital signs were as follows: blood pressure of 108/58 millimeters of mercury, heart rate of 138 beats per minute, respiratory rate of 23 breaths per minute, oral temperature of 37.4° Celsius, and a room air oxygen saturation of 86%. On physical exam, he appeared distressed, holding a container with approximately 150 milliliters (mL) of bloody sputum. He had bilateral conjunctivitis and was slightly icteric. His heart was tachycardic with a regular rhythm, and his heart sounds were normal. He was speaking in short sentences while actively coughing up blood and was tachypneic with diffuse rhonchi noted in all lung fields. His abdomen was soft and non-tender, with no organomegaly or masses. He had no joint swelling or signs of trauma or injury. His skin was warm and dry without any rashes or lesions. He was alert and oriented to person, place, time, and situation. Although distressed, he was cooperative and able to follow commands.

His initial labs (Table 1) showed several abnormalities including leukocytosis, anemia, hyponatremia, and elevated transaminases.

**Image 1:**
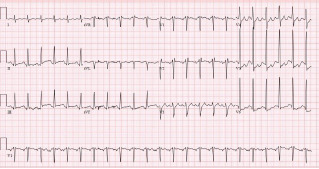
Electrocardiogram of a 44-year-old man with hemoptysis and hypoxemic respiratory failure showing sinus tachycardia with an incomplete right bundle branch block, left ventricular hypertrophy, possible left atrial enlargement, and marked ST abnormality, concerning for lateral ischemia.

**Image 2:**
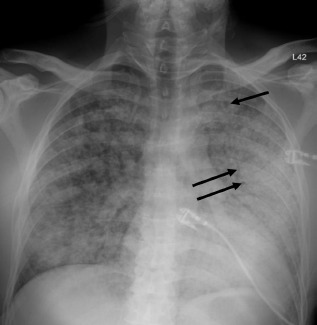
Portable chest radiograph of a 44-year-old man with hemoptysis and hypoxemic respiratory failure showing diffuse bilateral coalescent airspace opacities with air bronchograms (black arrows).

Unfortunately, there were no previous labs available for comparison. An electrocardiogram (ECG) was performed (Image 1), as well as a portable chest radiograph (Image 2). The patient was placed on a non-rebreather mask at a rate of 10 liters per minute.

## CASE DISCUSSION (DR. WILLIAMS)

Massive hemoptysis is a worrisome presentation, no matter the demographics of your patient. These patients often require emergent stabilization, which can limit the opportunity for a detailed history and even prohibit certain imaging options. This patient has a history of homelessness, polysubstance abuse, and likely minimal long-term outpatient medical care, which makes this case uniquely challenging from a diagnostic standpoint. It is a perfect setup for a rare etiology or an uncommon presentation. The starting differential for such a case is broad and includes a range of etiologies such as cardiovascular pathology, infections, malignancy, pulmonary sources, and traumatic injuries.^1^

Any diagnostic approach should begin with review of the existing information—history, exam, and any previous medical records. All of these may point in the direction of an underlying etiology for this patient’s hemoptysis. Summarizing the highlights of the case thus far, this patient has been suffering from fevers and myalgias for one week, followed by the development of a dry cough alongside some nausea and diarrhea. The patient ultimately sought medical attention due to coughing up blood. The exact quantity is poorly characterized, but I would argue this does not matter. While some older definitions of massive hemoptysis focus on rate and total volume of blood loss, the reality is it only takes 150 mL of liquid to fill the entirety of the conducting airways. This patient’s presentation should automatically be considered massive hemoptysis given his presenting respiratory failure and hemodynamic compromise.^1^

Ultimately, the symptoms detailed in the patient’s history are nonspecific at best and could point toward an infectious, malignant, or even autoimmune etiology. He presented during the COVID-19 pandemic, although he did not have any known exposure to the virus (or to tuberculosis). There is notably no history of trauma reported. This patient unfortunately did not have any previous medical records to offer additional clues.

Physical examination of this patient was concerning, although not necessarily helpful from a diagnostic standpoint. In rare situations, specific findings such as a hemangioma or telangiectasia can suggest a specific etiology such as an underlying vascular malformation. He had a low blood pressure, although not technically hypotensive, and significant tachycardia. This could represent hemorrhagic shock, sepsis, or consequences of his reported MDMA use. Additionally concerning is his room air oxygen saturation of only 86%. His diffuse rhonchi are more suggestive of a systemic or cardiovascular source as opposed to a specific hemorrhagic lesion, although exam alone cannot make this distinction. His noted conjunctivitis could further support an infectious etiology, while his mild icterus might suggest underlying liver disease and an associated coagulopathy.

Laboratory studies are vital in the work-up of hemoptysis but are rarely diagnostic in the ED. This patient’s workup revealed hyponatremia, presumed acute kidney injury, anemia, leukocytosis, and transaminitis. His D-dimer was also elevated. Hyponatremia may suggest a paraneoplastic syndrome, his renal failure could represent a vasculitis, and while a D-dimer is famously nonspecific, one must question the possibility of pulmonary embolism and/or malignancy in the setting of hemoptysis. It would have been potentially helpful to obtain some additional studies, although none of these would have likely led to a definitive diagnosis. (See the complete list of laboratory studies to consider in Table 2)

An ECG may similarly hold suggestive value but is ultimately unlikely to determine the etiology of a patient’s hemoptysis. This patient demonstrated sinus tachycardia with an incomplete right bundle branch block along with left ventricular hypertrophy (LVH) and possible left atrial enlargement. Tachycardia and right heart strain again suggest the possibility of pulmonary embolism, while the left atrial enlargement might suggest mitral valve stenosis as the cause of the hemoptysis. However, no murmur was noted on exam, thereby decreasing the probability that he had significant stenosis of the mitral valve, and pulmonary embolism should not cause diffusely abnormal lung sounds as were heard in this patient. Left ventricular hypertrophy could hint at congestive heart failure, although this is a rare cause of hemoptysis and even rarer for it to present so severely.

This leads me to the patient’s imaging, which is often key to reaching a final diagnosis in patients presenting with hemoptysis. As noted earlier, advanced imaging such as computed tomography (CT) is only possible once the patient has been sufficiently stabilized. Therefore, many physicians may have to initially rely on a portable chest radiograph (CXR) alone. A CXR is a bit of a mixed bag, with widely variable rates of diagnostic ability reported in the literature. Sometimes it can identify a localizing lung lesion such as a tumor or cavitation, or potentially a more diffuse process such as pneumonia or diffuse alveolar hemorrhage. This patient’s CXR fits more in the latter category with diffuse bilateral airspace disease and air bronchograms. It is fortunate that he was also able to receive a CT angiography (CTA), as this is often the ideal imaging study to identify the cause of bleeding in hemoptysis, especially if it is from a culprit lesion. As most cases of massive hemoptysis are from the bronchial arterial system, it is worth noting that this CTA should ideally be protocoled differently from the traditional pulmonary artery CTA used when evaluating for pulmonary embolism.^1^ This patient’s post-intubation CTA demonstrated widespread ground-glass opacities with prominent septal lines, dependent consolidation, and air bronchograms in the bilateral lung bases suggestive of diffuse alveolar hemorrhage and hemorrhagic pneumonia.

Point-of-care bronchoscopy is the only other major diagnostic tool to consider in the evaluation of massive hemoptysis. Availability, however, is inconsistent, and its diagnostic yield is generally lower than that of CT. It would not seem to have much value in this patient, although it could have been considered had the patient been too unstable for transport to a CT scanner.

So, what does this all mean in terms of an underlying diagnosis? There are several ways to categorize the diagnostic possibilities. I previously listed some of the major categories for massive hemoptysis: cardiovascular, infection, malignancy, pulmonary, and traumatic. Others have suggested breaking the differential diagnosis for reported hemoptysis into the following categories: source other than the lower respiratory tract; tracheobronchial source; pulmonary parenchymal source; primary vascular source; and miscellaneous/rare causes.^2^

At this point, a cardiovascular etiology seems unlikely. The CTA did not identify any vascular malformations or culprit lesions. The symptoms are too profound for mitral valve stenosis, and there was no significant coagulopathy within the patient’s labs. The CT imaging similarly appears to exclude malignancy and any traumatic injury, and there was no history to suggest the latter. This leaves me focused on pulmonary and infectious etiologies of this patient’s presentation.

This patient unquestionably has diffuse alveolar hemorrhage, but the lingering question of “why” remains. Some possibilities include autoimmune diseases such as granulomatosis with polyangiitis, systemic lupus erythematous, and Goodpasture syndrome. While all three of these can produce an alveolar hemorrhage syndrome, I would have expected more significant and consistent additional features of autoimmune diseases such as arthralgias, rashes, or urinary symptoms. This leaves me predominantly considering an infectious source.

Hemorrhagic pneumonia can be viral (eg, hantavirus, Ebola, Lassa virus), fungal (eg, mycetoma), or bacterial. Bacterial infections may include Mycobacterium (eg, tuberculosis), typical and atypical bacteria, as well as rickettsial and zoonotic disease. This patient presented during the cooler months of the year in the mid-Atlantic, which makes ehrlichiosis, anaplasmosis, or Rocky Mountain spotted fever all unlikely from an epidemiologic standpoint, given the low risk of tick exposure during this window of time. This leads me to consider some of the more infrequently diagnosed bacterial infections and their potential environmental or zoonotic origins.

The patient’s presentation includes a constellation of features that fit with a couple of less common infections. Legionella presents with hyponatremia, alveolar hemorrhage, conjunctivitis, and hemoptysis. Legionella is classically contracted through inhalation or aspiration of contaminated water. There is no such mechanism of exposure reported in the patient’s history. Leptospirosis, however, also has a case to be made here. Classically it has an initial phase from 2–9 days with fever and viral symptoms and produces several physical exam findings exhibited by this patient—conjunctival insufflation and scleral icterus. Furthermore, the patient has risk factors secondary to his social determinants of health. He is currently homeless in a city known for its significant rodent population. Brown rats, the most common type in Baltimore, are a known significant natural reservoir for the virus. In the second phase of leptospirosis, patients can demonstrate liver damage and bleeding. The most severe form is referred to as Weil disease and would explain the patient’s acute respiratory distress and altered mental status.

While both diagnoses seem possible, an incidental and unexplained exposure to Legionella simply seems less plausible than exposure to leptospirosis given the known risks from the brown rat population in the patient’s city. Therefore, I think leptospirosis is the most likely diagnosis. It should be confirmed using a urine test for leptospirosis DNA or a serum test for immunoglobulin M antibodies.

## CASE OUTCOME (DR. MCNEILLY)

The patient was admitted to the medical intensive care unit (MICU) where he remained intubated for several days. An echocardiogram was performed, which was overall unremarkable, including no sign of mitral valve disease or depressed left ventricular ejection fraction. A bronchoscopy was performed, which showed no signs of inhalation injury, and serial aliquots showed successive clearing, inconsistent with diffuse alveolar hemorrhage. A bronchoscopic culture was obtained that grew methicillin-sensitive *Staphylococcus aureus* (MSSA). The patient’s blood cultures had been negative up to that point; so his antibiotic regimen was narrowed to cefazolin to cover MSSA pneumonia. Despite continued treatment, the patient’s clinical condition and ventilator requirements did not improve.

Infectious disease (ID) was consulted for a suspected spirochetal infection. Given the time of year (April), ID had a low suspicion for tick-borne illnesses, including ehrlichiosis or Lyme disease. Previous studies had demonstrated a significant reservoir of leptospirosis in the city’s rat population, and there had been numerous rainstorms in the weeks leading up to the patient’s presentation, along with intermittent flooding of the streets. Urine and serum samples were sent for polymerase chain reaction testing and did, in fact, return positive for leptospirosis. He was started on ceftriaxone for severe leptospirosis. After a 10-day stay in the MICU, he was transferred to the medicine floor and then left the hospital against medical advice. He has since presented to the hospital for unrelated complaints, with no apparent sequelae from his bout of leptospirosis.

## RESIDENT DISCUSSION

As the most widespread zoonotic disease in the world, leptospirosis is of global importance.^3^ The incidence of leptospirosis is higher in the tropics and occurs in both industrialized and developing countries. *L. interrogans*, the causative organism of leptospirosis, is a highly motile obligate anaerobic spirochete with features of both Gram-positive and Gram-negative bacteria.

Leptospirosis typically follows a biphasic pattern, starting with an influenza-like bacteremia phase during which the leptospires are circulating in the blood. The acute bacteremia phase is followed by the immune phase, during which leptospiral toxins result in an immune-mediated response.^4^ Clinical manifestations of leptospirosis range from an influenza-like illness (anicteric form) to fulminant disease, known as Weil disease.^5^–^7^ Weil disease is characterized by a classic triad of jaundice, renal impairment, and hemorrhages. It carries a mortality rate of 5–15%.^8^–^10^ Pulmonary hemorrhages are increasingly recognized as a major and potentially lethal complication of leptospirosis. Pulmonary involvement occurs in up to 70% of severe cases and predicts a poor outcome in which death can occur within 48 hours.^11^,^12^

Leptospires are carried in the proximal renal tubules of animals, with human infections resulting from exposure to the urine of carrier animals, either directly or from contaminated soil or water.^3^ In both rural and urban areas, rats are a major carrier of leptospires. In a study of rats in one American urban center, 65.3% were found to carry antibodies against *Leptospira interrogans*.^13^ Given that most cases of leptospirosis are transmitted indirectly via contaminated water,^14^ the large reservoirs of leptospirosis carried by rats pose a significant threat during floods in both rural and urban settings, particularly to dwellers of lower socioeconomic means.^15^,^16^

Indeed, a recent study conducted in 2021 showed that transmission rates of leptospirosis depend on both flooding and temperature. Given the increasing frequency of extreme weather events in the setting of continued global warming, there may be an upsurge in the incidence and magnitude of leptospirosis outbreaks around the world.^17^ In addition, ongoing climate change may lead to increasing prevalence in regions such as the United States that have thus far experienced fewer cases.

Most cases of leptospirosis are self-limited and resolve without antimicrobial therapy. Prophylactic dosing in some individuals living or traveling in endemic areas may be beneficial.^18^ Hawaii and Puerto Rico are the most common geographic location in the US for leptospirosis, although cases are also identified throughout the world. It is unclear whether treatment in mild disease limits the progression to severe disease.^19^–^22^ A systematic Cochrane review found that antimicrobial therapy did not affect mortality in mild cases; however, there was a nonsignificant trend toward expedited resolution of illness.^23^ In general, if symptoms are significant enough to come to clinical attention, and the diagnosis is suspected, the patient should receive antimicrobial treatment.

For mild disease, a seven-day course of doxycycline, or a three-day course of azithromycin, is recommended. These courses also cover rickettsial infections, which can have a similar presentation. Patients with severe disease should be treated with a seven-day course of intravenous (IV) penicillin, doxycycline, ceftriaxone, or cefotaxime. These patients will likely have some degree of organ failure requiring supportive care, which is generally managed the same as organ failure associated with other etiologies of sepsis. Unique therapies that have been proposed include IV corticosteroids, given the vasculitic nature of the disease process; however, additional studies are needed to support their efficacy.^24^,^25^ While plasmapheresis has also been proposed, high-quality data is lacking on its efficacy.^26^

## FINAL DIAGNOSIS

Severe leptospirosis (Weil disease)

## KEY TEACHING POINTS

1. Leptospirosis is the most widespread zoonotic disease in the world, and cases may continue to rise as a result of the increased flooding associated with climate change.
2. Weil disease is a severe manifestation of leptospirosis, and classically presents as a triad of jaundice, renal impairment, and hemorrhage.
3. Pulmonary hemorrhage occurs in up to 70% of patients with Weil disease and portends a poor outcome in which death can occur within 48 hours.

## Figures and Tables

**Table 1:** Laboratory results of a 44-year-old man with hemoptysis and hypoxemic respiratory failure.

Blood test	Patient value	Normal range
Complete blood count		
White blood cells	15.3 K/mcL	4.5–11.0 K/mcL
Hemoglobin	8.7 g/dL	12.6–17.4 g/dL
Hematocrit	25.5%	37.0–50.0%
Platelets	147 K/mcL	153–367 K/mcL
White blood cell differential		
Neutrophils	87.0 %	42.6–74.5 %
Lymphocytes	3.9 %	20.8–50.5 %
Monocytes	3.3 %	2.0–10.3 %
Basophils	0.5 %	0.2–1.0 %
Eosinophils	0.1 %	0.9–2.9 %
Serum chemistries		
Sodium	128 mmol/L	136–145 mmol/L
Potassium	3.7 mmol/L	3.5–5.1 mmol/L
Chloride	96 mmol/L	98–107 mmol/L
Bicarbonate	19 mmol/L	21–30 mmol/L
Blood urea nitrogen	55 mg/dL	9–20 mg/dL
Creatinine	1.91 mg/dL	0.66–1.25 mg/dL
Glucose	94 mg/dL	70–99 mg/dL
Calcium	8.0 mg/dL	8.6–10.2 mg/dL
Magnesium	1.4 mg/dL	1.6–2.6 mg/dL
Phosphorous	4.9 mg/dL	2.5–4.5 mg/dL
Total protein	6.3 g/dL	6.3–8.2 g/dL
Albumin	2.8 g/dL	3.5–5.2 g/dL
Lactate	1.3 mmol/L	0.5–2.2 mmol/L
Hepatic studies		
Total bilirubin	2.0 mg/dL	0.3–1.2 mg/dL
Aspartate aminotransferase	154 u/L	17–59 u/L
Alanine aminotransferase	93 u/L	0–49 u/L
Alkaline phosphatase	117 u/L	38–126 u/L
Cardiac studies		
N-terminal prohormone of brain natriuretic peptide	1,420 pg/mL	<300 pg/mL
Troponin	<0.02 ng/mL	<0.06 ng/mL
Coagulation studies		
Prothrombin time	14.5 seconds	12.1–15 seconds
Partial thromboplastin time	37 seconds	25–38 seconds
International normalized ratio	1.1	0.8–1.1
D-dimer	1,570 ng/mL FEU	<499 ng/mL FEU
Urine Studies		
pH	5.0	5.0–8.0
Protein	Negative	Negative
Ketones	Negative	Negative
Bilirubin	Negative	Negative
Urobilinogen	0.2 mg/dL	0.1–1.8 mg/dL
Nitrites	Negative	Negative
White blood cells	3–5 per hpf	0–5 per hpf
Red blood cells	0–2 per hpf	0–2 per hpf
Bacteria	Negative	Negative

*K*, thousand; *mcL*, microliter; *g*, gram; *dL*, deciliter; *mmol*, millimole; *L*, liter; *mg*, milligram; *u*, units.

*ng*, nanogram; *mL*, milliliter; *FEU*, fibrinogen equivalent units; *mg*, milligram; *dL*, deciliter; *hpf*, high power field.

**Table 2:** Laboratory studies to consider in the work-up of massive hemoptysis.

Laboratory test
Complete blood cell count
Basic metabolic panel
Renal and liver function
Prothrombin time and international normalized ratio
Blood type
Blood antibody screen
Fibrinogen level
Thromboelastrography (TEG)
Rotational thromboelastogram
Sputum culture
Blood culture
